# Major Causative Bacteria of Dairy Cow Mastitis in the Inner Mongolia Autonomous Region, China, 2015–2024: An Epidemiologic Survey and Analysis

**DOI:** 10.3390/vetsci12030197

**Published:** 2025-02-21

**Authors:** Hongmei Zhao, Ting Guo, Yaping Zhou, Fengmiao Zhao, Yajie Sun, Yuchen Wang, Yuchen Bian, Guangyuan Tian, Chunxia Wu, Qi Cui, Xue Zhou, Jinlei Cui, Han Si, Yongqing Hao

**Affiliations:** 1 College of Veterinary Medicine, Inner Mongolia Agricultural University, Hohhot 010018, China; hongmeizhao@emails.imau.edu.cn (H.Z.);; 2 Inner Mongolia Academy of Agricultural and Animal Husbandry Sciences, Hohhot 010031, China; 3 Inner Mongolia Tongliao Agricultural and Animal Husbandry Science Research Institute, Tongliao 028000, China; 4 Inner Mongolia Hulunbuir Animal Disease Control Center, Hulunbuir 021000, China

**Keywords:** cattle, mastitis, pathogens, environmental, contagious

## Abstract

In this study, we sought to evaluate the prevalence of bacterial pathogens of mastitis in dairy cattle in the Inner Mongolia Autonomous Region, China. The study was conducted from 2015 to 2024 using a total of 12,053 clinical mastitis (CM) and sub-clinical mastitis (SCM) samples. The pathogens were isolated and identified by standard bacteriological and mycological methods. The most common pathogens isolated were *Escherichia coli* (13.82%), *Staphylococcus aureus* (10.28%), *Klebsiella* spp. (8.96%), *Streptococcus agalactiae* (7.45%), *Streptococcus uberis* (6.60%), *coagulase-negative staphylococci* (5.84%), and *Streptococcus dysgalactiae* (4.21%). From 2015 to 2017, the primary pathogens responsible for causing mastitis in cows were *Staphylococcus aureus* and *Streptococcus agalactiae*. In 2018, the most frequently isolated pathogen was *Staphylococcus aureus*. Notably, the isolation rate of *Escherichia coli* increased from 12.31% to 21.72%, and the isolation rate of *Klebsiella* spp. increased from 7.52% to 14.01% from 2019–2024. Mycoplasma was only detected in clinical mastitis cases, with a separation rate as high as 6.95%. In summary, the isolation rate of environmental pathogens is gradually increasing, while that of contagious pathogens has been continuously declining. This indicates that the current prevention strategies for infectious pathogens are effective. As a next step, it will be important to develop new strategies specifically targeting environmental pathogenic microorganisms.

## Simple Summary

This study investigated the type, percentage, and epidemiological trend of pathogenic bacteria of mastitis in dairy cows in large intensive farms in the Inner Mongolia Autonomous Region. We collected 12,053 mastitis milk samples from 20 large dairy farms in Inner Mongolia, China from 2015 to 2024. Bacterial isolation and identification were performed on the mastitis milk samples. *Escherichia coli*, *Staphylococcus aureus*, *Klebsiella* spp., *Streptococcus agalactiae*, and *Streptococcus uberis* were the most commonly isolated bacteria, while *Mycoplasma* was only common in clinical mastitis. Environmental pathogens will likely represent a future challenge in controlling mastitis in dairy cows. This has led to the development of rational control measures for local large-scale intensive dairy farms, which have greatly reduced the incidence of mastitis. The study provides a reference for the prevention and control of mastitis in dairy cows worldwide.

## 1. Introduction

Mastitis is a disease that poses significant harm, is associated with a significant financial burden, and is difficult to prevent and treat on dairy farms. Globally, mastitis has long been an important disease that has plagued the health of the dairy industry [1]. Mastitis is not only a significant cause of milk yield loss but also reduces the nutritional value and quality of milk, and can even endanger human health, severely impacting the economic benefits of dairy cow farming. Infection by pathogenic microorganisms is the main cause of mastitis in dairy cows, and more than 137 species of pathogenic bacteria have been found to be associated with mastitis in dairy cows, although only approximately 20 of these species are commonly found [2]. Numerous studies have reported on the main types and prevalence of pathogenic microorganisms causing mastitis in dairy cows worldwide. A description of the global status of major pathogens responsible for causing mastitis in dairy cows indicates that *Staphylococcus aureus* (*S. aureus*) accounts for 25% of mastitis cases, followed by *coagulase-negative staphylococci* (*CoNS*) (20%), *Escherichia coli* (*E. coli*) (11%), *Streptococcus agalactiae* (*S. agalactiae*) (9%), and *Streptococcus uberis* (*S. uberis*) (9%) [3]. Bovine mastitis caused by *Prototheca* spp. infection is increasing steadily, a study demonstrates that *P. zopfii* gen. 2 is the third most common pathogen of mastitis in cattle in southeast Poland, with an overall incidence of 4.6% [4].

Mastitis can be categorized into clinical mastitis and subclinical mastitis based on its clinical manifestations. Clinical mastitis (CM) is characterized by visible abnormalities in the cow’s udder or milk. In affected cows, mastitis is often accompanied by systemic symptoms, elevated body temperature, depression, and loss of appetite. According to severity, it can be classified as mild, moderate, or severe. Subclinical mastitis (SCM) is the most common type of intramammary infection in cows with mastitis, and it is characterized by the absence of either localized or systemic clinical symptoms visible to the naked eye in the udder, as well as no change in the appearance of the milk [5,6]. However, subclinical mastitis can result in decreased milk production and increased somatic cell counts (SCCs), and requires a diagnostic test for detection, the most common of which is the California Mastitis Test (CMT) [6].

Mastitis can be classified into environmental mastitis and contagious mastitis based on the source of the pathogens. The main reservoir of environmental pathogens is the environment of dairy cows, while the main reservoir of contagious pathogens is the infected mammary area. There are many types of environmental pathogens, which primarily include Gram-negative bacteria such as *E. coli*, *Klebsiella*, *Serratia*, *Pseudomonas*, and *Proteus*, as well as Gram-positive, catalase-negative cocci such as environmental *streptococci*, *enterococci*, and *lactococci* [7]. The most common environmental mastitis pathogens among herds in North America are those grouped as coliforms and environmental *streptococci* [8].

The Inner Mongolia Autonomous Region of China, or Inner Mongolia for short, is located on China’s northern border, bordering Russia and Mongolia, with a total area of 1.183 million square kilometers. Inner Mongolia is an important production base for agricultural and livestock products in China. Animal husbandry is a distinctive and advantageous industry in Inner Mongolia, especially in terms of the number of dairy cows that are bred, which ranks first in China.

The Inner Mongolia grasslands cover 22% of the national grassland area. As an important national “milk tank”, in 2023, the region had 1.687 million dairy cows in stock and produced 7.926 million tons of milk, consistently ranking first in production in China [9]. Mastitis in cows severely restricts the development of dairy cow farming in Inner Mongolia, making it one of the most prevalent and critical diseases in the region. However, to the best of our knowledge, no previous study has conducted a comprehensive investigation and statistical analysis of the prevalence of CM and SCM and the prevalence of pathogens in dairy farms across various regions of Inner Mongolia. In cows, SCM is more challenging than CM, as SCM cases are often difficult to detect, and the infection rate can be 15–40 times higher than that of CM [10]. Therefore, the objective of this study was to conduct large-scale and long-term follow-up research to determine the main pathogenic bacterial species and prevalence of mastitis in dairy cows in Inner Mongolia. This will provide a solid foundation for the future prevention and control of mastitis in dairy cows in the Inner Mongolia region.

## 2. Materials and Methods

### 2.1. Recruitment of Herds

**Figure 1:**
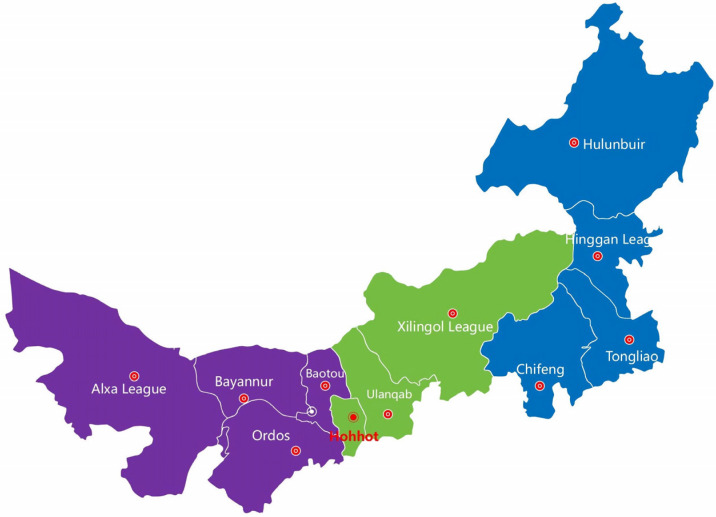
Geographic distribution of the tested dairy herds in the Inner Mongolia Autonomous Region. The numbers in the brackets represent the number of dairy farms, which are located below the names of the cities: Hohhot (8), Baotou (3), Chifeng (3), Tongliao (2), Ordos (3), Hulunbuir (2), Bayannur (6), Ulanqab (2), Xing’an League (2), Xilingol League (2), and Alxa League (2).

In this study, mastitis milk samples were collected from 35 farms in the Inner Mongolia Autonomous Region between September 2015 and October 2024. Based on geographical location, these 35 ranches were selected from 227 intensive large-scale ranches (with more than 500 cattle) in Inner Mongolia. We sought to cover as much of the Inner Mongolia region as possible. A total of 12,053 mastitis milk samples were collected from lactating cows with CM (*n* = 4632) and SCM (*n* = 7421) from dairy farms (*n* = 35) located in the following 11 leagues and cities: Hohhot (8/64), Baotou (3/22), Chifeng (3/21), Tongliao (2/10), Ordos (3/20), Hulunbuir (2/10), Bayannur (6/59), Ulanqab (2/10), Xing’an League (2/4), Xilingol League (2/3), and Alxa League (2/4) (Figure 1).

All veterinarians and farm workers who participated in the survey and inspected the pasture had undergone training at the Microbiology and Immunology Laboratory of the Veterinary Medicine College of Inner Mongolia Agricultural University and followed the relevant sampling rules of this laboratory. During the study period, the Microbiology and Immunology Laboratory of the Veterinary Medicine College of Inner Mongolia Agricultural University offered free mastitis milk sample testing services to all participating farms in the survey research, along with providing corresponding prevention and treatment plans to encourage farmers to actively cooperate with the survey research.

### 2.2. Sample Collection

#### 2.2.1. Clinical Mastitis Collection

CM cases are generally discovered by the farm veterinarian in charge during their regular daily work. These cases are identified by clinical symptoms of mastitis, such as redness, swelling, heat, pain, hard lumps, or nodules within the udder, or changes in the characteristics of the milk (such as blood in the milk, thin watery milk, clots in the milk, or flocculent precipitates). Before starting antibiotic treatment, the veterinarian or workers collected milk samples from the affected clinical mastitis quarters.

Collectors were instructed to collect milk samples using the following procedure: first, clean disposable gloves were worn, and the teats were wiped with a sterilized warm towel. Then, an iodine preparation was applied as a medicinal bath, ensuring that the medicinal bath time was no less than 30 s. After the medicinal bath, the teats were dried, and the first three foremilks were discarded to exclude contaminated stray bacteria, and the fourth milk was collected into a sterilized 50 mL collection tube. The centrifuge tubes were then labeled with the cow number, lactation area, and date of sampling.

#### 2.2.2. Sub-Clinical Mastitis Collection

Sub-clinical mastitis milk samples are collected by screening with the CMT assay. CMT testing is performed once a year on each partner farm. Farms randomly select 50–100 healthy cows for this test. The procedure for sterilizing the udder before sampling was the same as for CM sample collection. After disinfection, 2 mL of milk samples from each quarter were placed into four cups on the testing tray. An equal amount of commercial CMT reagent was then added to each cup. The mixture was gently swirled in a circular motion on a horizontal plane for 10 s. CMT reagent can cause cell lysis, which releases DNA and proteins, thereby increasing the viscosity of the mixture. This increase in viscosity indicates a rise in the somatic cell count, which is visually assessed through a 5-point scoring system: 0 (negative, the mixture remains liquid with no visible precipitation), trace (mild precipitation that disappears with continuous stirring), 1+ (mild reaction, with distinct precipitation but no gel formation), 2+ (moderate reaction, the mixture thickens immediately and forms some gel), and 3+ (strong reaction, forming a distinct gel that adheres to the bottom of the stirrer). Milk samples with CMT scores of 0 and trace were recorded as negative. In this study, milk samples were collected from udder areas with a CMT score of ≥1+ for sub-clinical mastitis [11].

Mastitis milk samples collected from farms that were close to the laboratory were brought back for testing on the same day using a sampling box. For pastures that were further away, the milk samples were first placed in a −20 degree freezer, before being sent via cold chain express delivery to the Laboratory of Microbiology and Immunology at the Veterinary Medicine College of Inner Mongolia Agricultural University for testing as quickly as possible. All participating ranches had pre-established long-term cooperative relationships before the initiation of this project, ensuring the continuity of sampling.

### 2.3. Microbiological Culture and Identification

Upon arrival at the laboratory, all frozen samples were thawed at room temperature. The milk samples were cultured for pathogenic microorganisms using the standard methods described in the National Mastitis Council (NMC) guidelines [12]. Briefly, after mixing the milk sample, a calibrated inoculation loop was used to collect 2–3 loops of milk sample, which were then streaked in a Z-pattern on a blood agar plate (Haibo, Qingdao, China). The plates were incubated aerobically at 37 °C for 24 to 48 h. The samples were considered to be culture positive if one or more colonies were observed (≥100 cfu/mL). If no pathogenic microorganisms were observed after 48 h of continued culture, the sample was considered to be free of pathogenic microorganisms. Samples yielding two bacterial species were grouped as “mixed culture”, whereas samples yielding three or more bacterial species were considered to be contaminated.

**Figure 2:**
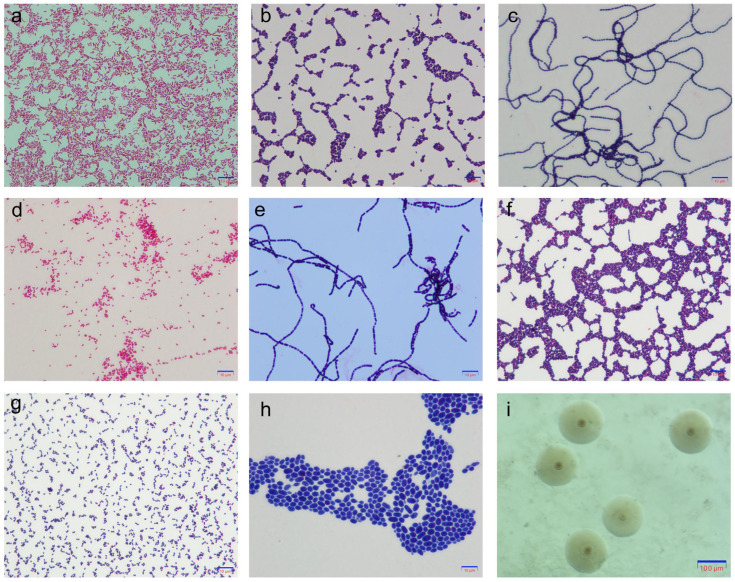
(**a**) *Escherichia coli*, Gram stain negative, non-spore, straight bacilli with bluntly rounded ends, scattered or in pairs. (**b**) *Staphylococcus aureus*, Gram stain positive, rounded without spores, arranged in grape bunches. (**c**) *S. agalactiae*, Gram stain positive, round or ovoid, often in long chain chains. (**d**) *Klebsiella pneumoniae*, Gram stain positive, short thick straight bacilli, arranged singly, in pairs or in short chains. (**e**) *Bacillus cereus*, Gram stain positive, rod-shaped, mostly in chains, ends bluntly rounded, spore-producing. (**f**) *Lactococcus garvieae*, Gram stain positive, round or oval, short chains or pairs, no spores. (**g**) *Streptococcus lutetiensis*, Gram stain positive, round, short chain-like arrangement. (**h**) *Saccharomyces cerevisiae*, Gram stain positive, larger oval shaped, arranged in clusters. and (**i**) *Mycoplasma bovis* observed under an inverted microscope, colonies of *Mycoplasma bovis* on solid medium with “scrambled eggs”-like appearance with a dark central umbilicus, round, and transparent.

Isolates were identified using growth characteristics on blood agar, by microscopic appearance after staining (Gram and Giemsa), and through biochemical testing. We identified that *S. aureus*, *S. agalactiae*, *S. dysgalactiae*, *S. uberis*, and *E. coli.*, *S. aureus* form round, large colonies on blood agar plates, with diameters ranging from 0.5 mm to 1.5 mm. These colonies are grayish or milky-white, and may be α-hemolytic, β-hemolytic, or non-hemolytic. *S. aureus* is Gram-positive and produces grapelike cluster-forming cocci (Figure 2b). Moreover, a positive hydrogen peroxide test, a positive rabbit plasma coagulase test, and detection of the *nuc* gene are indicative of *S. aureus* [13]. Streptococci appear as round, gray-white or opalescent, transparent or semi-transparent small colonies, or colonies as tiny as pinpoints on blood agar plates. Streptococci are Gram-positive, with a round or oval shape and exist in pairs, clusters like grapes, or chain-like arrangements (Figure 2c). Streptococci appear as a negative result on the hydrogen peroxide test and are differentiated by whether they are esculin-positive (e.g., *S. uberis*) or esculin-negative (e.g., *S. dysgalactiae* and *S. agalactiae*) [14]. The Christie–Atkins–Munch-Petersen (CAMP) test is used to differentiate between CAMP-negative *S. dysgalactiae* and CAMP-positive *S. agalactiae* [15]. *E. coli* and other Gram-negative bacteria grow into round, raised colonies on blood agar plates; they are translucent, gray-white, with a diameter of 2–3 mm. *E. coli* are Gram-negative non-spore-forming bacilli, with blunt ends, and are scattered or arranged in pairs (Figure 2a). *E. coli* is identified by performing motility tests, indole reaction, ornithine decarboxylase test, oxidase test, and growth on triple sugar iron slants [16].

### 2.4. Mycoplasma Culture

A small amount of milk sample was inoculated onto *Mycoplasma* solid medium (1 gL^−1^ glucose, 0.4 gL^−1^ sodium pyruvate, 21 gL^−1^ pleuropneumonia-like organism (PPLO) broth, 10 mL 25% (*w*/*v*) yeast extract, 2.5 mL 0.4% (*w*/*v*) phenol red, 200 mL donor equine serum [Hyclone; Logan, UT, USA], 1% (*w*/*v*) agar, 10 mL 10,000 IU mL 1 penicillin-G, and pH 7.6–7.8 adjusted with 0.5 mol L^−1^ sodium hydroxide) [17] using a sterile loop in a sterile environment, before incubating at 37 °C in a 5% CO_2_ incubator for 3 to 7 days. After the completion of *Mycoplasma* culture, pinhead-sized transparent colonies were observed on the culture medium. Under the microscope (Olympus SZX7, Tokyo, Japan), suspicious colonies appeared like fried eggs, with a “central navel” in the center, varying in size, and with smooth edges (Figure 2i).

### 2.5. Yeast Culture

The samples were inoculated in duplicate on Sabouraud chloramphenicol dextrose agar (SDA) (Haibo, Qingdao, China) and incubated aerobically at 25 °C for 48–72 h. The yeasts were identified using colony morphology and microscopic examination [18]. Colonies grew as white, circular-shaped, and Gram-positive. Under the microscope, large colonies appeared blue, with water-drop shaped arrangements (Figure 2h). In bovine mastitis, fungi are usually *Candida albicans* and *Saccharomyces cerevisiae*.

### 2.6. Molecular Biology Identification

The confirmation of most pathogenic species requires molecular biological methods. We performed pure culture on the isolated pathogens and then extracted bacterial genomic DNA. The extracted bacterial genomic DNA was used as a template for polymerase chain reaction (PCR) amplification of the target fragment, using the universal 16S rRNA primers 27F/1492R. For the identification of fungal pathogens, we used the universal primers ITS1/ITS4. The primer sequences were synthesized by Shanghai Sangon Biotech Company (Shanghai, China) (Table 1), and the PCR products were submitted to Shanghai Sangon Biotech for sequencing and identification.

### 2.7. Statistical Analysis

A database of the clinical types of mastitis, year, pathogen species, and quantity was established using Microsoft Excel 2020. All statistical analyses were conducted using SPSS v29.0 (SPSS Inc., Chicago, IL, USA). Statistical evaluation was performed using the chi-square test. A *p*-value of < 0.05 was considered statistically significant.

## 3. Results

### 3.1. Culture Results

We collected a total of 12,053 mastitis milk samples from 35 large-scale dairy farms in the Inner Mongolia Autonomous Region from September 2015 to October 2024 (2015–2024, *n* = 1101, 1206, 1223, 1135, 1210, 1131, 1234, 1198, 1280, and 1335, respectively). Notably, 86.35% (10,408/12,053) of the milk samples tested positive for pathogenic bacteria. Of these, the positive rate of pathogens in CM samples was 86.40% (4002/4632), while that in SCM samples was 86.32% (6406/7421). Additionally, 10.10% (1217/12,053) of the mastitis milk samples showed no growth of pathogens, whereas 3.55% (428/12,053) of the mastitis milk samples were determined to be contaminated. (Table 2).

### 3.2. Comparison of Pathogens Detected in CM and SCM Samples

In the total mastitic milk samples collected over a decade, *E. coli*, *S. aureus*, and *Klebsiella* spp. were the most commonly isolated, followed closely by *S. agalactiae*, *S. uberis*, and CoNS. The isolation rate of *E. coli* in CM samples was 16.67% (772/4623), followed by *Klebsiella* spp. at 11.55% (535/4623), *S. aureus* at 10.82% (501/4623), and *S. agalactiae* at 7.15% (331/4632). In SCM samples, the isolation rate of *E. coli* was 12.05% (894/7421), followed by *S. aureus* at 9.94% (738/7421), and *CoNS* at 8.76% (650/7421). The isolation rates of *S. agalactiae* and *Klebsiella* spp. in SCM samples were 7.64% (567/7421) and 7.34% (545/7421), respectively. *E. coli* and *Klebsiella* spp. are significantly more common in CM than in SCM samples (*p* < 0.05). The isolation rates of *S. aureus*, *S. agalactiae*, and *S. dysgalactiae* in CM and SCM were not significantly different (*p* > 0.05). *S. uberis* and CoNS are more commonly found in SCM, significantly higher than in CM samples (*p* < 0.05).

*Mycoplasma* pathogens were detected in only 6.95% (322/4632) of CM samples, *Corynebacterium* was detected in 3.89% (289/7421) of SCM samples, and the *Corynebacterium* were only detected in SCM samples.

Pathogenic bacteria of the Enterococcus genus are mainly isolated from SCM samples, with an isolation rate of 5.50% (408/7421), while they only account for 0.93% (43/4632) of CM samples. *Pseudomonas aeruginosa* was mainly isolated in CM samples, with an isolation rate of 4.92% (228/4632), while in SCM, the isolation rate was only 0.94% (70/7421). The isolation rates of other Enterobacteriaceae and Lactococcus species pathogens were higher in SCM samples than in CM milk samples (*p* < 0.05).

The isolation rates of *Proteus*, *Serratia*, and *Yeast* in CM were higher than that in SCM samples (*p* < 0.05). The isolation rate in CM milk samples was 3.41% (158/4632), 3.20% (148/4632), and 3.39% (157/4632), while those in SCM milk samples were 1.77% (131/7421), 1.64% (122/7421), and 0.86% (64/7421), respectively.

Other *Streptococcus* spp. were identified in 2.44% (113/4632) of CM samples and 2.82% (209/7421) of SCM samples, with no significant difference (*p* > 0.05).

**Figure 3:**
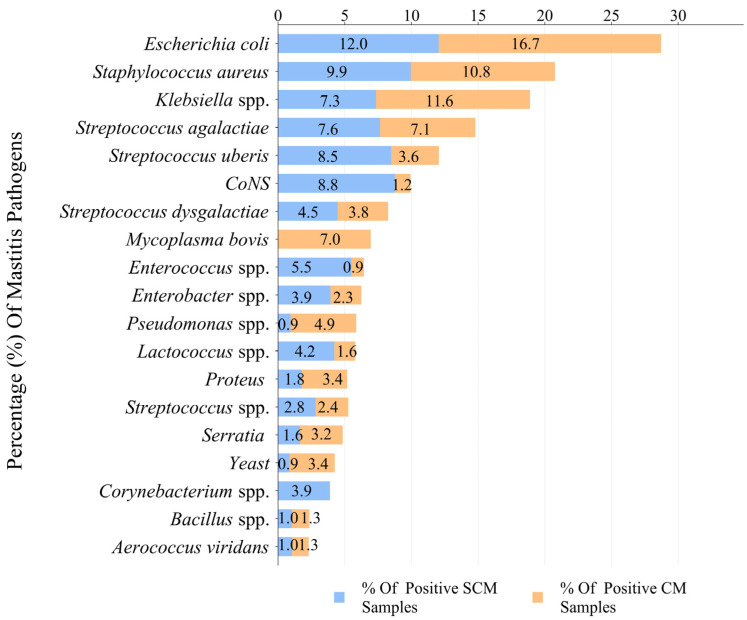
Comparison of pathogens detected in milk samples from cattle with CM and SCM. CM: Clinical mastitis, SCM: Sub-clinical mastitis, *CoNS*: *Coagulase-negative Staphylococci*.

Of all the collected mastitis milk samples, *Bacillus cereus* and *Aerococcus viridans* had the lowest pathogen isolation rates (Figure 3).

### 3.3. Comparison of Pathogens Detected in Environmental Mastitis and Contagious Mastitis

From 2015 to 2017, *S. aureus* and *S. agalactiae* were the main pathogens causing clinical and subclinical infections, and the isolation rates increased annually. From 2015 to 2017, the isolation rates of *S. aureus* were 16.98%, 17.74%, and 18.81%, respectively. The isolation rates of *S. agalactiae* were 11.81%, 12.27%, and 13.33%, respectively. The isolation rate of *E. coli* also increased slowly, with rates of 6.63%, 7.30%, and 8.67% respectively over the 3 years. The isolation rate of *Klebsiella* spp. also showed an increasing trend over these 3 years, with rates of 3.27%, 4.48%, and 5.72%, respectively. Between 2015 and 2017, the isolation rates of *S. aureus* and *S. agalactiae* were higher than those of *E. coli* and *Klebsiella* spp.

In 2018, the isolation rate of *S. aureus* remained the highest at 14.27%. However, it decreased by 2.71%, 3.47%, and 4.53% compared to the rates from 2015 to 2017, respectively. In 2018, the isolation rate of *S. agalactiae* was 9.96%, which, for the first time, was lower than the isolation rate of *E. coli* at 10.66%. Starting from 2018, the isolation rate of *E. coli* has been continuously increasing. At the same time, in 2018, the isolation rate of *Klebsiella* spp. was 6.52%, which was higher than those in the previous 3 years.

**Figure 4:**
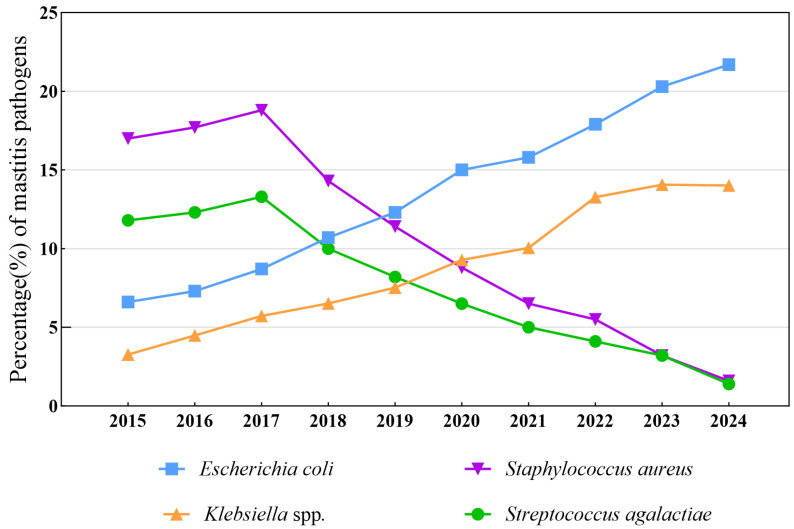
Trends in the major pathogens causing mastitis in dairy cows in the Inner Mongolia Autonomous Region from 2015 to 2017.

From 2019 to 2024, environmental pathogens represented by *E. coli* and *Klebsiella* spp. became the main pathogens causing infectious mastitis. In 2019, the isolation rate of *E. coli* was 12.31%, which increased to 21.72% by 2024. The isolation rate of *Klebsiella* spp. was 7.52% in 2019 and reached 14.01% in 2024. From 2019 to 2024, the isolation rate of *S. aureus* decreased from 11.40% to 1.65%, while that of *S. agalactiae* decreased from 8.18% to 1.42%. By 2024, the isolation rates of both *S. aureus* and *S. agalactiae* were less than 3% (Figure 4).

The chi-square test was used to determine differences in the isolation rates of each pathogen between the years 2015 and 2024. A *p*-value < 0.05 was indicative of statistically significant differences. The results show that 84.21% (16/19) of pathogens exhibited significant differences between different years. The isolation rates of *E. coli*, *Klebsiella* spp., *S. aureus*, *S. agalactiae*, *S. dysgalactiae*, *Pseudomonas* spp., *Enterobacter* spp., *Mycoplasma*, *Aerococcus viridans Serratia*, and other *streptococci* were extremely significant (*p* < 0.001) across different years. The isolation rates of *Proteus*, *Bacillus* spp., *Corynebacterium*, *Strept. uberis*, and *Lactococcus* spp. were significantly different among different years (*p* < 0.05), whereas those of *Enterococcus* spp., *CoNS*, and *Yeast* showed no significant differences over the 10 year period (*p* > 0.05) (Table 3).

## 4. Discussion

Both CM and SCM can lead to the significant loss of milk production and a decline in milk quality. In this study, we found that *E. coli*, *S. aureus*, *CoNS*, *S. agalactiae*, *S. uberis*, and *Klebsiella* spp. were the most common pathogens in SCM milk samples, whereas, in CM milk samples, *E. coli*, *Klebsiella pneumoniae*, *S. aureus*, *S. agalactiae*, and *Mycoplasma bovis* were the most common. The prevalence of CM and SCM in dairy cows in Pakistan was 17% and 57%, respectively, from July 2018 to June 2019. The pathogenic bacteria isolated from mastitis milk samples were *Staphylococcus* spp. (34%), *E. coli* (19.4%), *Streptococcus* spp. (9%), and *Klebsiella* spp. (8%) [19]. In northeastern Poland, from 2013 to 2019, 1665 CM and SCM milk samples were isolated and identified, and *Streptococcus* spp. (39–49%) was identified as the most commonly isolated bacteria [20]. Moreover, in the Tierra Caliente region of Guerrero, Mexico, the prevalence of SCM was reported as being 20.5%, and 97.5% of pathogenic bacteria were identified as being Gram-negative. Approximately three-quarters of SCM cases are caused by *Proteus vulgaris, Salmonella* spp., and *Enterobacter aerogenes* [21].

Notably, the isolation rates of *E. coli* and *Klebsiella* spp. significantly increased in both the SCM and CM milk samples, showing a trend of annual growth. The numbers of other environmental pathogens, such as *S. agalactiae*, *CoNS*, *Pseudomonas aeruginosa*, *Serratia* spp., and *Proteus* spp., are also continuously increasing. Furthermore, in North America between 2011 and 2022, the four main pathogens isolated from mastitic milk samples from 29 veterinary laboratories were *S. agalactiae*, *S. uberis*, *S. aureus*, and *E. coli* [22]. Moreover, the mastitis research team from China Agricultural University selected 15 large-scale dairy farms from 12 major milk-producing provinces in China to investigate the types and isolation rates of mastitis pathogens. As a result, the most commonly isolated pathogens were *Staphylococcus* (39.03%), *Streptococcus* (11.01%), *Bacillus* (8.24%), *Pseudomonas aeruginosa* (6.76%), and *Acinetobacter* (3.38%), the majority (67.53%) of which are environmental pathogens [23]. Investigation into the pathogenic microorganisms responsible for causing mastitis in dairy cows, both domestically and internationally, has revealed that the prevalence of these pathogens varies by region. To prevent and control mastitis in dairy cows, it is important for each region to conduct local epidemiological surveys and analyze the main pathogenic microorganisms of mastitis in dairy cows.

In the past decade, these environmental pathogens have not only increased in number but have also become more diverse in species. The *Aerococcus viridans* isolated in this study is an example of a pathogenic species that has been discovered in recent years. The increasing prevalence of environmental pathogens causing mastitis in dairy cows, coupled with a decreasing incidence of infectious pathogens, is consistent with findings from related surveys in other countries [24]. There are likely two main reasons behind this phenomenon. First, China has implemented strict regulatory measures, requiring that the somatic cell count in milk from large-scale farms does not exceed 200,000 cells/mL. This standard is significant for identifying the prevalence of sub-clinical mastitis in cattle herds, especially that caused by infectious pathogens. Second, farms have implemented prevention and treatment strategies against infectious pathogens and have strengthened farm management, including by implementing regular maintenance of milking equipment, and adherence to standard operating procedures such as post-milking teat dipping. The proper execution of these measures has effectively reduced the infection rate of mastitis caused by infectious pathogens.

*Mycoplasma bovis* is a relatively common pathogenic *Mycoplasma* species found in bovine mastitis [25]. In this study, the isolation rate of *Mycoplasma bovis* in clinical mastitis milk samples was as high as 6.95%. *Mycoplasma bovis* is a contagious pathogen that can spread rapidly among cattle herds and causes mastitis with a long course of disease that can last for months. *Mycoplasma* is rarely found in SCM, and in this study, *Mycoplasma bovis* was not isolated from SCM mastitis milk samples, only detected in CM, which is consistent with the report by Maid Rifatbegovi [26]. *Mycoplasma bovis* is prevalent in bovine mastitis in most dairy farms worldwide [27]. In the Inner Mongolia region, *Mycoplasma bovis* can cause acute mastitis in cattle herds, where infected cows experience a rapid decrease in milk production, leading to the complete cessation of lactation. The milk turns yellowish brown and has a serous-purulent consistency. The infection often spreads from one mammary quarter to several. Clinical rehabilitation is slow, and even if recovery is achieved, it is difficult to determine whether true healing has occurred. The outbreak of *Mycoplasma* mainly occurs because *Mycoplasma* is not regularly checked. Our research team suggests that farms conduct regular screening for *Mycoplasma*. An investigation from South Australia revealed the prevalence of *Mycoplasma bovis* to be 6.2% [28], which is similar to the results of this study. However, it is much lower than the 75% in the United States [29], and the morbidity rate of *Mycoplasma bovis* in Japan is 3.8% [30]. These differences are likely related to the number of samples evaluated in each study and other factors. The infection rate of *Mycoplasma* mastitis in dairy cows in the Inner Mongolia Autonomous Region has been increasing annually from 2015 to 2024, partly due to the continuous introduction of new, untested *Mycoplasma* cattle each year. Additionally, the increasing maturity of *Mycoplasma* detection technology in recent years has made regular screening of *Mycoplasma* in herds easier for farms to achieve. With the screening and control of *Mycoplasma* pathogens, we anticipate a gradual reduction in the cases of mastitis in cows caused by *Mycoplasma* in the future.

In the dairy farms of China, compared to previous investigations on mastitis, the distribution of infectious pathogens, mainly *S. aureus* and *S. agalactiae*, has undergone many changes. Although both remain highly prevalent pathogens based on our results, the trend suggests that infectious pathogens will not be the main epidemic pathogens of mastitis in the future. Conversely, environmental pathogens such as *E. coli*, *Klebsiella pneumoniae*, *S. uberis*, and *CoNS* will become problematic for mastitis. Contagious mastitis in dairy herds is controlled through post-milking teat dipping, comprehensive dry cow therapy, culling, treatment of clinical mastitis cases, and the proper maintenance of milking equipment. These measures are effective because they each help reduce the reservoirs of infectious pathogens within the herd. As the number of pathogen reservoirs decreases, the exposure of uninfected teats is reduced, new infections occur less frequently, and the reservoirs of infectious pathogens continue to diminish [31]. However, the effectiveness of prevention and control strategies for environmental pathogens is much more complex, largely owing to the fact that bacteria in the natural environment of dairy cows are diverse and unavoidable, such as in feces, bedding, and cowsheds. It is not possible to completely separate these from the dairy cows [32]. Developing new vaccines against environmental pathogens is urgently required. Moreover, the Inner Mongolia Autonomous Region has a relatively cold climate, so it is important to ensure good insulation, enhance nutrition, and improve animal welfare to also boost the immunity of dairy cows.

Another important reason for the increasing number of environmental pathogens in recent years is that the Chinese government has advocated for further strengthening environmental protection. Some pastures in the Inner Mongolia Autonomous Region have changed from using sand bedding to organic bedding materials such as sawdust, straw, and rice husks, which need to be frequently replaced and cleaned in the cowsheds. If not replaced in a timely manner, they can easily breed environmental pathogens such as *E. coli* and *Klebsiella pneumoniae*. Dairy cows on farms using organic materials had higher rates of mastitis from environmental pathogens such as coliforms and *Klebsiella* than dairy cows on farms using inorganic materials [33]. In a study conducted in Sweden, *Klebsiella* bacteria were associated with sawdust bedding, while *S. uberis* was more prevalent when straw or peat was used as bedding materials for cows [34]. Other studies have reported that first-lactation cows using new sand as bedding have a greatly reduced incidence of CM caused by Gram-negative bacteria compared to those using other organic bedding materials [35].

The increasing number of environmental pathogens and the gradual decline of infectious pathogens confirm the effectiveness of current prevention and treatment programs for infectious pathogens, but indicate that new strategies need to be developed to address environmental pathogens. Currently, there is no effective vaccine to prevent mastitis in dairy cows, therefore, it is necessary to implement regular monitoring of pathogen species. Monitoring pathogens that cause mastitis in cows will not only reduce the workload of farm veterinarians but also lower the clinical incidence of mastitis, thereby reducing economic losses on the farm.

Although we isolated, cultured, and analyzed mastitis-causing bacteria from large intensive dairy farms in the Inner Mongolia Autonomous Region over a 10 year period, this study still has several limitations. In recent years, we have noticed that many studies have mentioned the prevalence of *Prototheca* spp. in dairy cow mastitis [36]. However, we did not isolate *Prototheca* spp.; there are two possible explanations for this. Before 2016, we did not know enough about *Prototheca* spp. and it was often misdiagnosed as a fungal infection or missed due to sample contamination. From 2017 to 2024, we performed isolation cultures of the *Prototheca* spp., but the results were not isolated. Additionally, the Inner Mongolia Autonomous Region has a temperate continental climate, characterized by drought and little rain, a cold autumn and winter, and a windy spring and summer. These factors may lead to the lack of growth in *Prototheca* spp.

## 5. Conclusions

In this study, we investigated the main pathogens causing CM and SCM in 35 large-scale intensive dairy farms across 11 leagues in the Inner Mongolia Autonomous Region from 2015 to 2024. The primary pathogens were identified as *E. coli*, *Klebsiella pneumoniae*, *S. aureus*, *S. agalactiae*, *S. uberis*, and *CoNS*. *Mycoplasma* mainly causes clinical mastitis in dairy cows, and strengthening regular monitoring of *Mycoplasma* is an indispensable measure to reduce the incidence of infection. Additionally, environmental pathogens were confirmed as the main drivers of bovine mastitis in the Inner Mongolia Autonomous Region, providing a reliable basis for the prevention and treatment of mastitis in dairy cows in the Inner Mongolia Autonomous Region in the future.

## Figures and Tables

**Table 1:** Primer Sequences for Pathogen Identification.

Universal Primers	Primer Sequence	Annealing Temperature	Target Band(bp)
27F/1492R	F: 5′-AGAGTTTGATCCTGGCTCAG-3′	55 °C	1500
	R: 5′-TACCTTGTTACGACTT-3′		
ITS1/ITS4	F: 5-TCCGTAGGTGAACCTGCGG-3′	50.0 °C	800
	R: 5′-TCCTCCGCTTATTGATATGC-3′		

**Table 2:** Overview of the total number of dairy cow mastitis milk samples collected in the Inner Mongolia region from 2015 to 2024.

	Subclinical Mastitis					Clinical Mastitis					Total Mastitis				
Year	No. of Samples	No. of Positive Samples	Culture Negative	Contamination	No. of Positive Samples (%)	No. of Samples	No. of Positive Samples	Culture Negative	Contamination	No. of Positive Samples (%)	No. of Samples	No. of Positive Samples	Culture Negative	Contamination	No. of Positive Samples (%)
2015	728	622	83	23	85.44	373	326	22	25	87.40	1101	948	105	48.00	86.10
2016	798	683	101	14	85.59	408	358	26	24	87.75	1206	1041	127.00	38	86.32
2017	780	660	99	21	84.62	443	381	34	28	86.00	1223	1041	133	49	85.12
2018	712	617	84	11	86.66	423	364	31	28	86.05	1135	981	115	39	86.43
2019	749	640	90	19	85.45	461	396	32	33	85.90	1210	1036	122	52	85.62
2020	666	579	78	9	86.94	465	407	31	27	87.53	1131	986	109	36	87.18
2021	736	630	89	17	85.60	498	426	42	30	85.54	1234	1056	131	47	85.58
2022	702	612	79	11	87.18	496	426	44	26	85.89	1198	1038	123	37	86.64
2023	738	649	76	13	87.94	542	463	45	34	85.42	1280	1112	121	47	86.88
2024	812	714	90	8	87.93	523	455	41	27	87.00	1335	1169	131	35	87.57
Total	7421	6406	869	146	86.32	4632	4002	348	282	86.40	12,053	10,408	1217	428	86.35

**Table 3:** Significant differences in the prevalence of individual pathogens isolated from the milk samples of dairy cattle with clinical mastitis from 2015–2024.

Pathogen	*p*-Value
*Escherichia coli*	<0.001
*Klebsiella* spp.	<0.001
*Staphylococcus aureus*	<0.001
*Streptococcus agalactiae*	<0.001
*Streptococcus dysgalactiae*	<0.001
*Pseudomonas* spp.	<0.001
*Enterobacter* spp.	<0.001
*Mycoplasma*	<0.001
*Aerococcus viridans*	<0.001
*Serratia*	<0.001
Other *streptococci*	<0.001
*Proteus*	0.001
*Bacillus* spp.	0.001
*Corynebacterium bovis*	0.008
*Streptococcus uberis*	0.022
*Lactococcus* spp.	0.023
*Enterococcus* spp.	0.077
*CoNS*	0.163
*Yeast*	0.22

## Data Availability

Detailed data from the trial are available upon request.
